# Cost Analysis of the Use of Voriconazole, Posaconazole and Micafungin in the Primary Prophylaxis of Invasive Fungal Infections in Recipients of Allogeneic Hematopoietic Stem Cell Transplants

**DOI:** 10.36469/9832

**Published:** 2015-10-19

**Authors:** Santiago Grau, Carlos Solano, Carol García-Vidal, Isidro Jarque, Jon A. Barrueta, Carmen Peral, Irene Rodríguez, Darío Rubio-Rodríguez, Carlos Rubio-Terrés

**Affiliations:** 1 Hospital del Mar (IMIM), Barcelona, Spain; 2 Hospital Clínico Universitario, Valencia, Spain; 3 Hospital Universitari de Bellvitge, Barcelona, Spain; 4 Hospital Universitario La Fe, Valencia, Spain; 5 Pfizer S.L.U., Alcobendas (Madrid), Spain; 6 Trial Form Support, Madrid, Spain; 7 Health Value, Madrid, Spain

**Keywords:** cost-effectiveness analysis, micafungin, posaconazole, voriconazole, allogeneic hematopoietic stem cell transplant, prophylaxis, invasive fungal infections

## Abstract

**Objectives:** Compare the cost of the primary prophylaxis of invasive fungal infections (IFI) with voriconazole, posaconazole, and micafungin in patients undergoing allogeneic hematopoietic stem cell transplantation (HSCT) in hospitals of the National Health System (NHS) in Spain.

**Methods:** A cost analysis was made for 100 days and 180 days of prophylaxis and a decision tree model was developed. The efficacy rate of IFI prophylaxis and survival rate with liposomal amphotericin B treatment of prophylaxis failures were obtained from randomized trials and a meta-analysis of mixed treatment comparisons. The model simulation was interrupted with IFI treatment (prophylaxis failures). The costs of medication and its intravenous administration in the hospital (in the case of micafungin) were considered.

**Results:** In the non-modeled analysis, the savings per patient of prophylaxis with voriconazole ranged from €1,709 to €9,655 compared with posaconazole oral solution, from €1,811 to €9,767 compared with posaconazole gastro-resistant tablets and from €3,376 to €7,713 compared with micafungin. In the modeled analysis, the mean cost per patient of the prophylaxis and treatment of IFIs was €6,987 to €7,619 with voriconazole, €7,749 with posaconazole, and €22,424 with micafungin. Therefore, the savings per patient of prophylaxis with voriconazole was €130 to €3,664 and €11,132 to €30,374 compared with posaconazole and micafungin, respectively. The result remained stable after modification of the number of days of antifungal prophylaxis and the cost of antifungal treatment of failures.

**Conclusion:** Taking into account this model, antifungal prophylaxis with voriconazole in recipients of hematopoietic progenitor transplants, compared with posaconazole or micafungin, may represent savings for hospitals in Spain.

## INTRODUCTION

Invasive fungal infections (IFI) by yeasts and filamentous fungi are a persistent problem in Spain.[1] The widespread use of prophylactic fluconazole has resulted in a dramatic decline in the frequency of IFIs. However, IFIs due to filamentous fungi in general and invasive aspergillosis in particular continue to be a major health problem, especially in patients with acute leukemia and high-risk myelodysplastic syndromes, and in patients who have received allogeneic hematopoietic stem cell transplants (HSCT).[1,2] The incidence of proven or probable IFI due to filamentous fungi in hematological patients at risk ranges from 4% to 22%, in most cases involving invasive aspergillosis.[1] In addition, in hematology patients at risk the mean rate of mortality associated with invasive candidiasis is greater than 30% and that associated with invasive aspergillosis is greater than 50%.[1,3-5]

The cost of an episode of IFI in Spain has been estimated at €14,293. Out of this amount, 68% is due to the hospital stay and the remaining 32% is due mainly to the antifungal treatment.[6]

Given the health and economic impact of IFIs, it is important to carry out an adequate prevention strategy.[1] According to a recent meta-analysis of clinical trials of antifungal prophylaxis in patients undergoing HSCT, prophylaxis with voriconazole, micafungin, or posaconazole has been shown to be as effective, or more effective, than prophylaxis with fluconazole, the antifungal most often used in prophylaxis.[7]

The aim of this study was to estimate the cost of primary prophylaxis of the IFIs with voriconazole, posaconazole, or micafungin in patients undergoing HSCT from the perspective of the hospitals of the Spanish National Health System (NHS).

## METHODS

The effectiveness of IFI prophylaxis with voriconazole, posaconazole, and micafungin was assessed using two types of analysis.

### Non-modeled Analysis

The cost per patient of prophylaxis was assessed, taking into account the following variables: purchase exfactory price of the drugs[8], a treatment period of 100 days, or 180 days in the case of high risk patients[9], and, finally, patient body weight, which determines the daily dose of voriconazole and micafungin. For a body weight of more than 40 kg, the daily dose of voriconazole is 400 mg and that of micafungin is 50 mg. For a body weight of 40 kg or less, the daily dose of voriconazole is 200 mg and that of micafungin is 1 mg/kg.[9-12] It was assumed that after intravenous prophylaxis with micafungin patients would continue prophylaxis with oral voriconazole or posaconazole at discharge from the hospital.

### Modeled Analysis

A model decision tree was prepared in Microsoft Excel in which the three prophylaxis options were compared (voriconazole, posaconazole, and micafungin) in adults or children undergoing HSCT (Figure 1). The clinical trials from which the efficacy data were obtained were selected based on the following criteria: randomized design, use in primary IFI prophylaxis of patients undergoing HSCT, and comparisons with other antifungals. The efficacy rates of antifungals in the prophylaxis of IFIs were obtained from the clinical trials chosen, three involving comparisons to fluconazole[9,12,13] and one, comparisons to itraconazole[11] (Table 1). These studies were obtained through a systematic review of published comparative clinical trials of the three options compared. Antifungal prophylaxis was understood as efficacious in the absence of proven or probable IFI in the period set for the evaluation of efficacy in clinical trials: 4 weeks[12] with micafungin, 112 days[13] with posaconazole, or 100 to 180 days with voriconazole after HSCT[9,11] (Table 1). In the case of occurrence of IFI, the mortality due to HSCT or the underlying disease was increased by the mortality associated with IFI itself[14] (Table 1). Two types of costs were accounted: the cost of antifungal prophylaxis and IFI treatment costs in the event of prophylaxis failure. In the model, the cost of the medications and intravenous administration of IFI prophylaxis in the hospital (in the case of micafungin) was considered. The cost of antifungal treatment (€2014) was calculated from the laboratory market price (PVL)[8], the number of doses, the loading doses and the duration of prophylaxis considered in clinical trials[9,11-13,15] (Table 1). In addition to the cost of prophylaxis itself, the cost of prophylaxis failures was also estimated, i.e., the cost of antifungal treatment in patients in which IFI occurred despite prophylaxis (Figure 1). This cost, estimated at €10,515, was obtained from a recently published Spanish study in which it was assumed that if IFI occurred it was treated with liposomal amphotericin B.[16] All costs were updated to 2014 by the price index in Spain.

**Figure 1.** Decision Tree Model Presentation

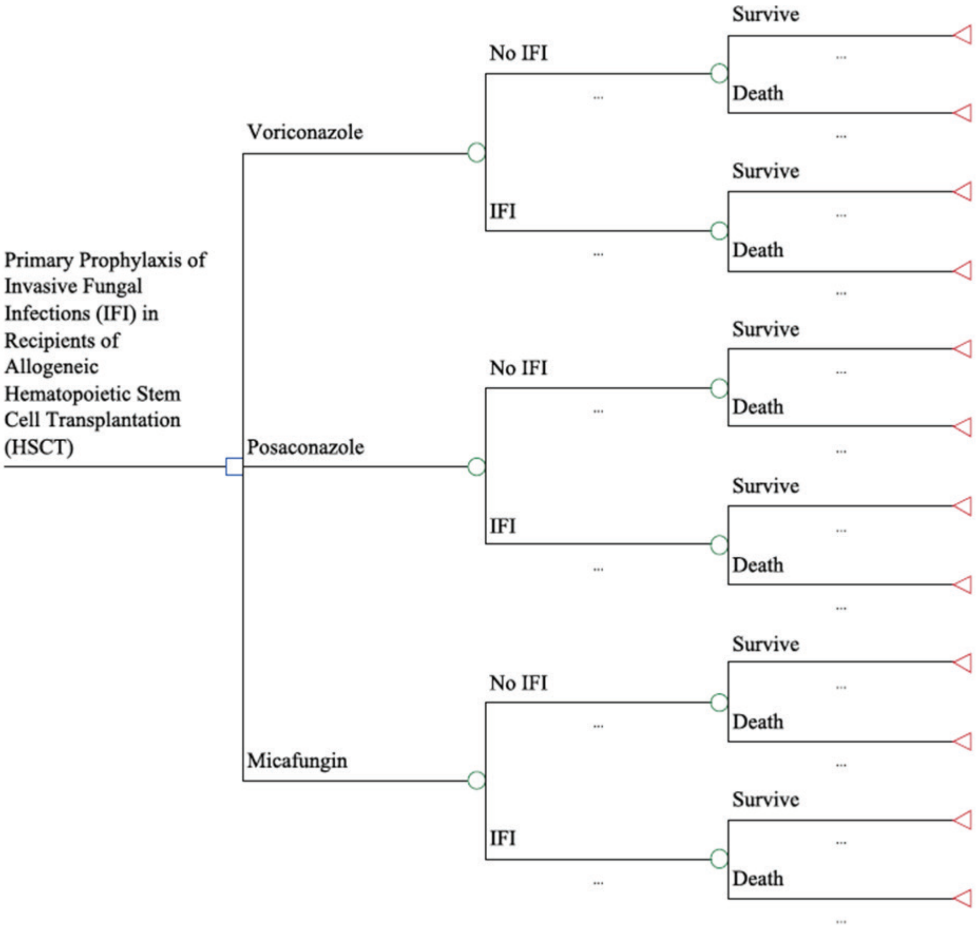

## Presentation of Results

The results of the non-modeled and modeled analyses were presented as the mean cost per patient of prophylaxis (and treatment of prophylaxis failures in the case of the model), and the difference in cost depending on the antifungal drug used. In the non-modeled analysis, the results obtained by changing the variables mentioned above (number of days of prophylaxis and body weight) were presented. Sensitivity analyses were made considering: the hypothesis that the vial for IV infusion of micafungin could be used in more than one patient in the event that only part of the vial contents was administered to a single patient[12]; discounts on the laboratory market price of voriconazole of 8%, 16%, and 24%; and analysis of the threshold sensitivity for possible discounts on the laboratory market price of posaconazole and micafungin. In the modeled analysis, a base case was analyzed using the mean values of all the variables, and deterministic sensitivity analyses were made in which the number of days of antifungal prophylaxis, cost of treating failures, and minimum and maximum costs were modified (Table 1).

**Table 1.** Variables and Assumptions of the Economic Model

| Item | Value/Assumption | References/ Sources |
|---|---|---|
| **IFI prophylaxis efficacy IFI [a]** | | |
| Voriconazole | 92.7% / 98.7% | 9,11 |
| Posaconazole | 94.7% | 13 |
| Micafungin | 80.0% | 12 |
| **Mortality from all causes in patients with / without IFI** | | |
| With IFI | 40.0% | 14 |
| Without IFI | 34.7% | 13,14 |
| **IFI prophylaxis dutation (days) [b]** | | |
| Voriconazole | 96 (34-101) | 9,11 |
| Posaconazole | 80 (64-96) | 13 |
| Micafungin | 18 (12-37) | 12 |
| **Drugs doses (mg/day)** | | |
| Voriconazole | 400 | 9,11 |
| Posaconazole | 600 | 13 |
| Micafungin | 50 | 12 |
| **Loading dose (first 24 hours)** | | |
| Patients weighing equal or superior to 40 kg | 400 mg every 12 hours | 15 |
| Patients weighing less than 40 kg * | 200 mg every 12 hours | 15 |
| **Drugs price (Ex-factory)[c]** | | |
| Voriconazole (Vfend 200 mg, 14 tablets) | €499.47 | 8 |
| Voriconazole (Vfend 200 mg, 28 tablets) | €998.92 | 8 |
| Posaconazole (Nofaxil 40 mg/ml, 105 ml oral solut.) | €629.09 | 8 |
| Posaconazole (Nofaxil 100 mg, 24 gastro-resistant tablets) | €720.00 | 8 |
| Micafungin (Mycamine 50 mg, 1 vial powder) | €214.19 | 8 |
| Micafungin (Mycamine 100 mg, 1 vial powder) | €428.57 | 8 |
| **Cost of IFI prophylaxis failure treatment** | | |
| Treatment with liposomic Anfotericin B [c,d] | €10 515 (€8412-12 618) | 16 |
| **Cost of intravenous prophylaxis with micafungin** | | |
| Inpatient intravenous infusion | €566.52 | 16 |
| % of infusions in hospital floor | 100% (0%) | Estimated |

IFI: invasive fungal infection *Patients with an age of 15 years or above
[a]Prophylaxis efficacy: not proven or probable IFI, in a period of 4 weeks[12], 112 days[13] or 180 days after PCT[9,11] [b] 95% confidence intervals [c]Costs for year 2014; [d] IV administration of liposomal amphotericin B, the daily dose of 3mg/kg, for a body weight of 70kg for 15 days[16] Range: ± 20%

## RESULTS

### Non-modeled Analysis

For 100 days of prophylaxis, the savings per patient treated with voriconazole compared with oral solution of posaconazole ranged from €1,709 to €5,348, depending on the scenario analyzed. When voriconazole was compared with gastro-resistant tablets of posaconazole (highest price that the oral solution) savings per patient treated with voriconazole ranged from €1,811 to €5,450. The savings per patient versus micafungin ranged from €3,376 to €5,560 depending on the scenario (Table 2).

**Table 2.** Non-modeled Costs Analysis Results

| A) Comparison of Voriconazole and Posaconazole (oral solution) | | | | |
|---|---|---|---|---|
| **Prophylaxis days** | **Body weight (kg)** | **Voriconazole cost per patient** | **Posaconazole cost per patient (oral solution)** | **Cost difference per patient** |
| 100 | ≥ 40 | €7279 | €8988 | - €1709 |
| 100 | < 40 | €3640 | €8988 | - €5348 |
| 180 | ≥ 40 | €13 045 | €16 178 | - €3133 |
| 180 | < 40 | €6523 | €16 178 | - €9655 |
| **B) Comparison of Voriconazole and Posaconazole (gastro-resistant tablets)** | | | | |
| **Prophylaxis days** | **Body weight (kg)** | **Voriconazole cost per patient** | **Posaconazole cost per patient (tablets)** | **Cost difference per patient** |
| 100 | ≥ 40 | €7279 | €9090 | - €1811 |
| 100 | < 40 | €3640 | €9090 | - €5450 |
| 180 | ≥ 40 | €13 045 | €16 290 | - €3245 |
| 180 | < 40 | €6523 | €16 290 | - €9767 |
| **C) Comparison of Voriconazole and Micafungin** | | | | |
| **Prophylaxis days** | **Body weight (kg)** | **Voriconazole cost per patient** | **Micafungin cost per patient** | **Cost difference per patient** |
| 100 | ≥ 40 | €7279 | €10 655* | - €3376 |
| 100 | < 40 | €3640 | €9200* | - €5560 |
| 100 | < 40 | €3640 | €8295** | - €4655 |
| 180 | ≥ 40 | €13 045 | €17 133* | - €4088 |
| 180 | < 40 | €6523 | €14 236* | - €7713 |
| 180 | < 40 | €6523 | €13 332** | - €6809 |

*No reutilization of vials for intravenous infusion. **With reutilization of vials for intravenous infusion.

For 180 days of prophylaxis, the savings per patient treated with voriconazole versus oral solution posaconazole ranged from €3,133 to €9,655, depending on the scenario analyzed. When voriconazole was compared with gastro-resistant tablets of posaconazole savings per patient treated with voriconazole ranged from €3,245 to €9,767. The savings per patient versus micafungin ranged from €4,088 to €7,713, depending on the scenario (Table 2). In the case that discounts on the laboratory market price for voriconazole of 8%, 16%, and 24% were given, the savings for a patient weighing 65 kg who received prophylaxis for 100 days would be €2,292, € 2,873, and €3,457, respectively, compared with oral solution of posaconazole, €2,393, €2,975 and €3,559, respectively, compared with gastro-resistant tablets of posaconazole, and €3,726, €4,075, and €4,425, respectively, compared with micafungin.

Prophylaxis with oral solution of posaconazole or micafungin would have a lower cost than voriconazole parting from discounts of 19% and 83% of the laboratory market price of posaconazole and micafungin, respectively.

### Modeled Analysis

The mean cost per patient of the prophylaxis and treatment of IFIs would be €6,987 to €7,619 with voriconazole, €7,749 with posaconazole, and €17,374 with micafungin, as can be seen in Table 3. Therefore, the savings per patient of prophylaxis with voriconazole would be €130 to €3,664 and €11,133 to €30,374 compared with posaconazole and micafungin, respectively. The result remained stable (savings with voriconazole versus posaconazole and micafungin) when the number of days of antifungal prophylaxis and cost of treating failure were modified (Table 3).

**Table 3.** Results of Deterministic Modeling Analysis (Base Case)

| **Voriconazole vs. Posaconazole** | | **Cost per patient** | |
|---|---|---|---|
| | **AVERAGE** | **MINIMUM** | **MAXIMUM** |
| Voriconazole (Wingard, 2010) | €7619 | €3040 | €8128 |
| Posaconazole (Ullmann, 2007) | €7749 | €6199 | €9299 |
| DIFFERENCE | - €130 | - €3159 | - €1,171 |

| **Voriconazole vs. Posaconazole** | | **Cost per patient** | |
|---|---|---|---|
| | **AVERAGE** | **MINIMUM** | **MAXIMUM** |
| Voriconazole (Marks, 2011) | €6987 | €2535 | €7371 |
| Posaconazole (Ullmann, 2007) | €7749 | €6199 | €9299 |
| DIFFERENCE | - €762 | - €3664 | - €1928 |

| **Voriconazole vs. Micafungin** | | **Cost per patient** | |
|---|---|---|---|
| | **AVERAGE** | **MINIMUM** | **MAXIMUM** |
| Voriconazole (Wingard, 2010) | €7619 | €3040 | €8128 |
| Micafungin (van Burik, 2004) | €22 424 | €14 173 | €37 745 |
| DIFFERENCE | - €14 805 | - €11 133 | - €29 617 |

| **Voriconazole vs. Micafungin** | | **Cost per patient** | |
|---|---|---|---|
| | **AVERAGE** | **MINIMUM** | **MAXIMUM** |
| Voriconazole (Marks, 2011) | €6987 | €2535 | €7371 |
| Micafungin (van Burik, 2004) | €22 424 | €14 173 | €37 745 |
| DIFFERENCE | - €15 437 | - €11 638 | - €30 374 |

## DISCUSSION

According to the present economic study, administering IFI prophylaxis to patients undergoing HSCT with voriconazole could generate savings for the hospitals of the National Health System of Spain.

In the assessment of these results, we should take into account both the potential limitations and consistencies of the study. With regard to the limitations, it must first be remembered that this is a theoretical model, which by definition is a simplified simulation of reality. In second place, a probabilistic analysis (Monte Carlo simulation) was not made due to the insufficiency of the data available for estimating the standard deviations of the variables.[17] In third place, it should be taken into account that the efficacy data from which the therapeutic success rates of the drugs compared were calculated originate from randomized clinical trials[9,11-13] and a meta-analysis.[14] However, the clinical trials did not directly compare the antifungals analyzed, so there is no absolute certainty that the efficacy data correspond to the same type of patients in terms of the prognostic factors. For example, in the studies of Wingard et al[9] and Ullmann et al[13], voriconazole and posaconazole, respectively, were compared with fluconazole. The cumulative incidence of IFI with fluconazole was also, respectively, 11.2% (95% CI, 7.7 to 14.7%) and 9.0%. This similarity in the response rate to prophylaxis with fluconazole might indicate some similarity between the patients in both studies. However, in the study of Ullmann et al[13] all the patients had graft-versus-host disease at baseline, in contrast with the patients enrolled in the study of Wingard et al.[9] Another possible weakness of the study could be the fact that it was not taken into consideration that, in clinical practice, part of the patients treated with voriconazole might receive it intravenously. In this sense, it should nevertheless be considered that the efficacy data used in the model come from clinical trials in which the treatments, both voriconazole[9,11] and posaconazole[13], were administered orally. Assuming that the same results would be obtained with the intravenous route in an undetermined percentage of patients adds uncertainty, due to the accumulation of ill-founded assumptions. Finally, it should be noted that the duration of prophylaxis with micafungin in the clinical trial was only 19 days on average[12], a much shorter duration than oral prophylaxis for 96 days with voriconazole[9,11] and for 80 days with posaconazole[13], which could have contributed to the lower rate of efficacy observed with micafungin in the clinical trial.

The study was limited to the costs of antifungal drugs and intravenous administration (in the case of micafungin). No other treatment costs such as hospital stay and adverse effects of drugs were considered. In this regard, it was assumed that these costs would be similar between treatments compared.

Regarding the consistencies of the study, the reliability of the result obtained, which was confirmed by sensitivity analysis, should be noted first.[18] In all the deterministic sensitivity analyses, voriconazole prophylaxis was what generated less costs per patient compared with posaconazole and micafungin. In second place, the follow-up of the patients who received prophylaxis with micafungin in the study of van Burick et al[12] was only four weeks (28 days) after 19 days of prophylaxis, for a total of 47 days. This followup was less than that carried out for voriconazole[9,11], which was 180 days. For this reason, it is possible that the efficacy results of micafungin may be overestimated when compared with voriconazole.

No economic analysis comparing voriconazole, posaconazole, and micafungin in the prophylaxis of IFIs in patients undergoing HSCT was identified. The effectiveness of the prophylaxis of IFIs with voriconazole compared to fluconazole was evaluated in a cost-effectiveness analysis conducted in the United States in patients who underwent HSCT.[19] According to this study, the added expense of prophylaxis with voriconazole rose to US $5,562 per patient, with a cost per year of life gained with voriconazole (the most effective prophylaxis) of $5,453. According to a probabilistic analysis, the probability of voriconazole prophylaxis being cost-effective in the United States in patients with HSCT would be 85% for an availability to pay $50,000 per year of life gained.

The effectiveness of the antifungal prophylaxis of IFIs with voriconazole has been analyzed in two Australian studies.[20,21] In a retrospective study published in 2010, in which an analysis was made of 56 and 38 patients with acute myeloid leukemia who received prophylaxis with voriconazole and posaconazole, respectively, the Monte Carlo simulation suggests that patients treated with posaconazole would generate less cost than with voriconazole, with a probability of 91.6%.[20] In a more recent retrospective study, 106 consecutive patients with acute myeloid leukemia who received prophylaxis with fluconazole, posaconazole, and voriconazole were analyzed. All the episodes of IFI (n = 3) occurred in the posaconazole group. On the other hand, patients with posaconazole prophylaxis had the highest rate of drug intolerance which led to the discontinuation of treatment (13% vs. 7% with fluconazole and voriconazole). A Monte Carlo simulation demonstrated a savings with fluconazole of 8,430 Australian dollars (AU $) (95% CI, AU $5,803 to 11,054) per patient compared with posaconazole and AU $3,681 (95% CI, AU $990 to 6,319) per patient compared with voriconazole.[21] Both Australian studies indicate possible savings with posaconazole versus voriconazole. However, these results are based on the efficacy data obtained in observational studies and they may be influenced by a retrospective design and, in any case, by the unit costs of health care resources in Australia.

Recently, a new drug form of posaconazole in tablets has been marketed at a higher price than the oral solution. No controlled, adequately powered clinical efficacy trial was conducted with the new formulation. Therefore, there is some uncertainty about the clinical efficacy of the new formulation of this drug.[22]

According to the results of this study, based on efficacy data from randomized clinical trials and the acquisition prices of antifungals in Spain, voriconazole may generate savings in Spanish hospitals compared with posaconazole and micafungin in the prophylaxis of IFIs in patients undergoing HCST.

## References

1 Vallejo JC, Ruiz-Camps I: Infección fúngica invasora en los pacientes hematológicos. *Enferm Infecc Microbiol Clin*. 2012; 30: 572–9.

2 Vallejo C, Len O: Infección fúngica invasora producida por Aspergillus spp. En: Pahissa A, editor. *La infección fúngica invasora*. Madrid: Entheos; 2010:161–83.

3 Kontoyiannis DP, Marr KA, Park BJ, et al: Prospective surveillance for invasive fungal infections in hematopoietic stem cell transplant recipients, 2001-2006: overview of the Transplant-Associated Infection Surveillance Network (TRANSNET) Database. *Clin Infect Dis* 2010;50:1091–100.

4 Lortholary O, Gangneux JP, Sitbon K, et al., French Mycosis Study Group: Epidemiological trends in invasive aspergillosis in France: the SAIF network (2005-2007). *Clin Microbiol Infect* 2011;17:1882–9.

5 Pagano L, Caira M, Nosari A, et al: Fungal infections in recipients of hematopoietic stem cell transplants: results of the SEIFEM B-2004 study—Sorveglianza Epidemiologica Infezioni Fungine Nelle Emopatie Maligne. *Clin Infect Dis* 2007;45:1161–70.

6 Peiró S, Gómez G, Rejas J, Guadarrama I, Blanca AB: Length of stay and antifungal treatments costs in patients with systemic mycosis: description and associated factors. *Value Health* 2002;5:564.

7 Ziakas PD, Kourbeti IS, Mylonakis E: Systemic antifungal prophylaxis after hematopoietic stem cell transplantation: a meta-analysis. *Clin Ther* 2014;36:292-306.

8 BOT Plus. Consejo General de Colegios de Farmacéuticos. BOT Plus 2.0. https://botplusweb. portalfarma. com/. Accessed June 16, 2015.

9 Wingard JR, Carter SL, Walsh TJ, et al: Blood and Marrow Transplant Clinical Trials Network. Randomized, double-blind trial of fluconazole versus voriconazole for prevention of invasive fungal infection after allogeneic hematopoietic cell transplantation. *Blood* 2010;116:5111-8.

10 Summary of Product Characteristics: Mycamine 100 mg, powder for solution for infusion. Micafungin sodium. http://www.aemps.gob.es/cima/pdfs/es/ft/08448002/FT_08448002.pdf. Accessed November 24, 2014.

[11] Marks DI, Pagliuca A, Kibbler CC, et al: IMPROVIT Study Group. Voriconazole versus itraconazole for antifungal prophylaxis following allogeneic haematopoietic stem-cell transplantation. *Br J Haematol* 2011;155(3):318-27.

[12] van Burik JA, Ratanatharathorn V, Stepan DE, et al: National Institute of Allergy and Infectious Diseases Mycoses Study Group. Micafungin versus fluconazole for prophylaxis against invasive fungal infections during neutropenia in patients undergoing hematopoietic stem cell transplantation. *Clin Infect Dis* 2004;39:1407-16.

[13] Ullmann AJ, Lipton JH, Vesole DH, et al. Posaconazole or fluconazole for prophylaxis in severe graft-versus-host disease. *N Engl J Med* 2007;356:335-47.

[14] Mills EJ, Perri D, Cooper C, et al: Antifungal treatment for invasive Candida infections: a mixed treatment comparison meta-analysis. *Ann Clin Microbiol Antimicrob* 2009;8:23.

[15] Summary of Product Characteristics: VFEND 50 mg, film-coated tablets. Voriconazol. http:// www.ema.europa.eu/docs/es_ES/document_library/EPAR__Product_Information/human/000387/WC500049756.pdf. Accessed November 24, 2014.

[16] De Salas-Cansado M, Echave M, Xue M, Charbonneau C, Barrueta A. Cost analysis of voriconazole versus liposomal amphotericin B for primary therapy of invasive aspergillosis among haematological patients in Spain. P1013. Poster presented at ECCMID, Berlin, Germany 27–30 April 2013. https://www.escmid.org/escmid_library/online_lecture_library/material/?mid=9374. Accessed June 30, 2014.

[17] Brigss A, Claxton K, Sculpher M. Decision modelling for health economic evaluation. Oxford: Oxford University Press; 2006.

[18] Rubio-Terrés C, Cobo E, Sacristán JA, Prieto L, del Llano J, Badía X, por el Grupo ECOMED: Análisis de la incertidumbre en las evaluaciones económicas de intervenciones sanitarias. *Med Clín* (Barc) 2004;122:668-74.

[19] Mauskopf J, Chirila C, Graham J, et al: Cost-effectiveness analysis of voriconazole compared with fluconazole for prevention of invasive fungal infection in patients receiving allogeneic hematopoietic cell transplants. *Am J Health Syst Pharm* 2013;70:1518–27.

[20] Al-Badriyeh D, Slavin M, Liew D, et al: Pharmacoeconomic evaluation of voriconazole versus posaconazole for antifungal prophylaxis in acute myeloid leukaemia. *J Antimicrob Chemother* 2010;65:1052-61.

[21] Heng SC, Slavin MA, Al-Badriyeh D, et al: Pharmacoeconomic evaluation of fluconazole, posaconazole and voriconazole for antifungal prophylaxis in patients with acute myeloid leukaemia undergoing first consolidation chemotherapy. *J Antimicrob Chemother* 2013;68:1669-78.

[22] Committee for Medicinal Products for Human Use (CHMP). Assessment report. Noxafil. International non-proprietary name: Posaconazole. Procedure No. EMEA/H/C/000610/X/0028. EMA/159150/2014. European Medicines Agency, 20 February 2014. Available at: http:// www.ema.europa.eu/docs/en_GB/document_library/EPAR_-_Assessment_Report_-_Variation/ human/000610/WC500168187.pdf. Accessed June 16, 2015.
